# Causes of Resistant Hypertension Detected by a Standardized Algorithm

**DOI:** 10.1155/2012/392657

**Published:** 2012-12-24

**Authors:** Livia Beatriz Santos Limonta, Letícia dos Santos Valandro, Flávio Gobis Shiraishi, Pasqual Barretti, Roberto Jorge da Silva Franco, Luis Cuadrado Martin

**Affiliations:** Departamento de Clínica Médica, Faculdade de Medicina de Botucatu, UNESP, Botucatu, SP, Brazil

## Abstract

Resistant hypertension (RH) is characterized by blood pressure above 140 × 90 mm Hg, despite the use, in appropriate doses, of three antihypertensive drug classes, including a diuretic, or the need of four classes to control blood pressure. Resistant hypertension patients are under a greater risk of presenting secondary causes of hypertension and may be benefited by therapeutical approach for this diagnosis. However, the RH is currently little studied, and more knowledge of this clinical condition is necessary. In addition, few studies had evaluated this issue in emergent countries. Therefore, we proposed the analysis of specific causes of RH by using a standardized protocol in Brazilian patients diagnosed in a center for the evaluation and treatment of hypertension. The management of these patients was conducted with the application of a preformulated protocol which aimed at the identification of the causes of resistant hypertension in each patient through management standardization. The data obtained suggest that among patients with resistant hypertension there is a higher prevalence of secondary hypertension, than that observed in general hypertensive ones and a higher prevalence of sleep apnea as well. But there are a predominance of obesity, noncompliance with diet, and frequent use of hypertensive drugs. These latter factors are likely approachable at primary level health care, since that detailed anamneses directed to the causes of resistant hypertension are applied.

## 1. Introduction

 Resistant hypertension (RH) is defined when blood pressure remains high (≥140 × 90 mm Hg) despite the use of three antihypertensive drug classes, including a diuretic and all prescribed in appropriate doses. In addition, the blood pressure controlled with the use of four or more medications is also considered RH. Since these patients are under greater risk of presenting reversible and/or secondary causes of hypertension, they may be benefited by therapeutical approach for this diagnosis. It is important to know the difference between arterial hypertension (AH), noncontrolled, and RH because the first one includes patients with low adherence to treatment or under inappropriate treatment.

 Although its prevalence is not known for sure, it is a common clinical problem mainly due to the increase in life expectancy and the prevalence of chronic diseases such as diabetes, obesity, and kidney disease. According to the VI Brazilian Guidelines of AH [1], a Brazilian study revealed that 59.1% of adult individuals are known to be hypertensive; 67.3% of them were in treatment and only 26.1% had their blood pressure controlled (140 × 90 mm Hg). The same source also highlights that approximately 70.6% of deaths in 2007 in Brazil were a result of cardiovascular disease and stroke which was the leading cause of death throughout the country [1].

 The AH is an important risk factor to the development of cardiovascular diseases and complications like cerebral vascular disease, coronary artery disease, heart failure, chronic kidney disease and peripheral vascular disease. In this context, it is estimated that 40% of the deaths by stroke are secondary to AH, as well as 25% of the deaths by coronary artery disease. The prognosis of patients with RH compared with patients with AH easily controlled was specifically evaluated in a recent study that demonstrated a higher cardiovascular risk among RH patients [2]. However considering the significant reduction of the risk achieved with the effective treatment of AH, it is estimated that the effective control of RH promotes more significant benefits.

 It is a condition of multifactorial origin including genetic factors, lifestyle, use of certain drugs, and secondary causes, namely, obstructive sleep apnea, primary hyperaldosteronism, Cushing's syndrome, pheochromocytoma, renal parenchymal disease, renal artery stenosis, hypothyroidism, hyperthyroidism, acromegaly, hyperparathyroidism, and aortic coarctation [2].

 As has been shown in the literature and in medical practice, the RH is a relevant clinical problem found in the general population. Given the risk that the maintenance of very high blood pressure represents for the development of diseases with significant mortality, it is assumed that the RH increases the susceptibility to these conditions, exposing the resistant hypertensives to a higher risk. Furthermore, in this set of patients, we can identify a unique opportunity to prevent diseases associated with AH. However, the RH is currently little studied, is necessary and more knowledge of this clinical condition in order to improve the prognosis of its bearers. In addition, this is a study of resistant hypertension in a Brazilian population that is an emergent country. These reports from this kind of population are uncommon in the literature. 

 In view of the assumptions made above, we proposed the analysis of specific causes of RH by using a standardized protocol in Brazilian patients diagnosed in a center for evaluation and treatment of hypertension. 

## 2. Methods

**Figure 1:**
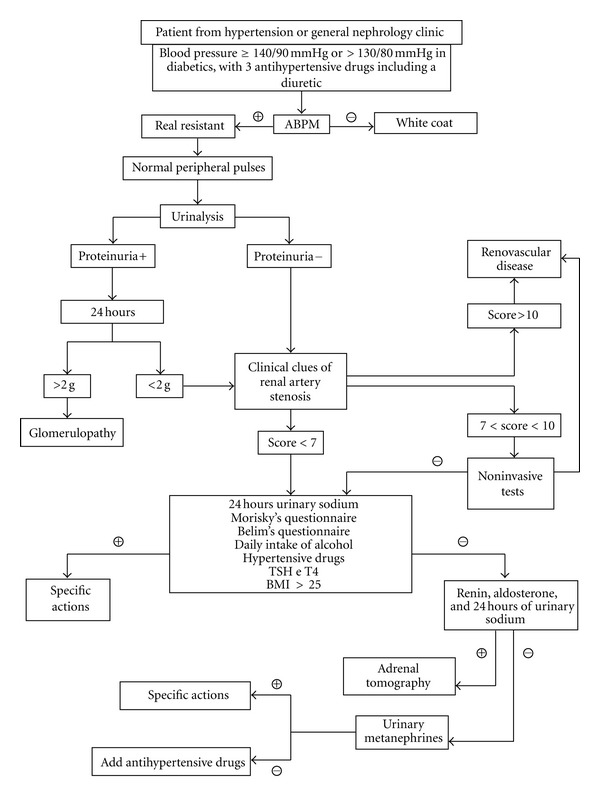
Fluxogram for treatment of resistant hypertension.

 The current study has monitored patients diagnosed with resistant hypertension treated by a university center for management of AH (CHA) in Botucatu Medical School (Botucatu SP Brazil). The management of these patients was conducted in an outpatient resistant hypertension clinic linked to CHA, with application of a preformulated protocol which aimed at the identification of the causes of resistant hypertension in each patient through the management standardization (Figure 1). The clinical protocol was composed of.

- Ambulatory blood pressure monitoring (ABPM) to the exclusion of pseudo-resistance by white coat effect.
- Measurement of arterial stiffness by evaluation of aortic pulse wave velocity, central blood pressure measurement, and augmentation index.
- Analysis of proteinuria urinary sediment and estimation of glomerular filtration rate to check the presence of parenchymatous renal disease.
- Perform a clinical score of renovascular disease [3].
- Verification of lifestyle factors: quantifying 24 hours urinary sodium; calculation of body mass index (BMI); survey of hypertensive drugs use; application of questionnaires of compliance (Morisky survey) [4] and obstructive sleep apnea (Berlin questionnaire) [5–7]; reminder of daily intake of alcohol and application of CAGE questionnaire [8, 9].
- TSH and T4 dosage for search of hyper and hypothyroidism.
- Determination of renin, aldosterone, and adrenal tomography for the investigation of primary aldosteronism when applicable.

When identified the source of resistance, the necessary adjustments were made in the treatment of these patients aiming the at improvement of their condition.

## 3. Statistical Analysis 

 The discrete variables are expressed as a percentage. Continuous variables of parametric distribution were expressed as mean ± standard deviation and of nonparametric distribution in median (first quartile and third quartile).

## 4. Results

 Eighty-six out of 209 patients from the outpatient hypertension clinic had resistant hypertension criteria, and 30 of them agreed to participate in this study.

 These thirty patients assessed were composed by seven male and twenty-three females, of whom six afrodescendants and twenty-four whites. The age was 55 ± 11.9 years. The patients had a weight of 84 ± 21.1 kg and 162 ± 11.0 cm high, resulting in a BMI of 32 ± 5.6 kg/m^2^, 36 of them had a BMI over and 30 kg/m^2^.

 The ABPM systolic blood pressure (SBP) was 145 ± 19.9 mm Hg, and diastolic blood pressure (DBP) was 87 ± 13.4 mm Hg. The office SBP was 147 ± 19.4 mm Hg, while the office DBP was 14.1 ± 90 mm Hg. The sleep SBP was 139 ± 22.4 mm Hg, and the sleep DBP was 11.8 ± 79 mm Hg. The dip of SBP was 0.05 ± 0.10, and the dip of DBP was 0.11 ± 0.10 during sleep. The percentage of nondipper patients was 31% dipper for SBP and was 44% dipper for DBP.

 Used antihypertensive classes were calcium channel blockers in 64%; converting enzyme inhibitors in 63% of patients; angiotensin II antagonist in 43%; converting enzyme inhibitors or angiotensin II antagonist or aliskiren in 96%; beta-blockers in 36%; alfa-2 adrenergic agonist in 36%; spironolactone in 32%; vasodilators in 11%; aliskiren in 0.04%. All patients were using diuretics during the study.

 The Morisky questionnaire pointed out a frequency of 68% patients not compliant to treatment. After the beginning of this study, all the patients became adherent to the drug treatment, but only two of them had achieved an adequate blood pressure control. So, only two patients had pseudoresistance, which shows the representativeness of our approach. The application of Berlin questionnaire pointed out a frequency of 44% patients with high risk of obstructive sleep apnea, and they were referred for expert evaluation. Eighty percent of the patients presented either low compliance or high risk for sleep apnea. The application of CAGE questionnaire, referring to use of alcohol, obtained only a positive response.

 Eight patients used hypertensive drugs four of these used chronically diclofenac, and one of them associated sibutramine with amfepramone. Among patients who made use of hypertensive drugs, two had low adherence and high risk for sleep apnea.

 The score for renovascular disease was 8.67 ± 2.54 points. Two patients presented a score higher than 10 points in the Renovascular disease score; these patients were referred to hemodynamic assessment. One of these patients presented a significant atherosclerotic disease in left renal artery. A patient had score less than 7, but the ultrasound showed renal asymmetry. This patient was confirmed as having renal vascular disease in hemodynamic assessment.

 Among the laboratory tests performed, the 24 hours urinary was 204.41 ± 98.79 mEq/24 h. The excretion of sodium in 24 hours was above 150 mEq/24 h in 53% of patients, and only one patient presented sodium excretion less than 100 mEq/24 h.

 The urinalysis pointed a proteinuria frequency of 7 in 30 patients. The 24 hours proteinuria was 0.35 ± 0.66 g/24 h, one patient had proteinuria exceeding 2 g. This patient had all clinical criteria for diabetic nephropathy, and proteinuria remitted after tight control of blood pressure with intensive inhibition of renin angiotensin axis. The median of albuminuria was 20 (5–523), and the prevalence of albuminuria >30 mg/24 h was 36%. All patients with albuminuria > 30 mg/24 h were nondipper or to SBP or to DBP.

 TSH was 2.56 ± 1.79 mUI/L. Two patients had TSH above the limit of normality. In two patients, the aldosterone (ng/dL)/renin (ng/mL/h) relationship was over 30, and only one showed an abnormality in adrenal tomography (hyperplasia). One patient had a reduction in serum potassium with normal aldosterone/renin relationship and responded satisfactorily to the use of amiloride. This patient was diagnosed as having Liddle's syndrome.

 One patient presented a hematocrit of 68% and hemoglobin of 22 g/dL and was referred to hematological investigation, and an analysis of bone marrow confirm the diagnosis of Polycythemia vera. After proper treatment, the blood pressure levels reduced.

 So, seven patients had secondary hypertension of different causes; 19 of the 23 remaining patients failed to reducing the blood pressure values by obstructive sleep apnea, high sodium intake, and a low compliance to drug treatment in the beginning of protocol. These patients became adherent in the followup with a improvement in blood pressure. Nevertheless only two patients of these achieved blood pressure target. Only four patients could not find any obvious cause for resistant hypertension. All patients were evaluated as outpatients from the proposed protocol, and the appropriate therapeutics were implemented.

## 5. Discussion

 Resistant hypertension is a serious public health problem, and its treatment has sparked attention from experts in hypertension. Identifying their causes has a fundamental importance. Few Brazilian studies conduct a systematic investigation of these causes. These data bring the information about the causes of resistant hypertension in a hypertension clinic of an emergent country and are very uncommon in the literature. In this way, this knowledge could encourage other studies in the same fashion. Furthermore, few studies used an algorithm for differential diagnosis in resistant hypertension highlighting the importance of our approach. There are many revisions or studies that identify only hyperaldosteronism. However, there are few studies that apply a standardized and broad algorithm and show the results as we do in this paper.

 In this study, were identified seven patients with secondary resistant hypertension in 30 patients, two patients with primary hyperaldosteronism, renovascular disease in two, one with a renal parenchymatous disease, a Liddle's syndrome, and a Polycythemia vera in one. Almost all the 23 patients had some degree of noncompliance either to drug treatment or diet. However, after the beginning of the study, all patients became adherent and were using effectively at least 3 medications, but only two of them had achieved blood pressure control.

 Thirty patients of 86 in the resistant hypertension criteria agreed to participate in the implementation of the protocol and accompaniment; however, even with the limited number of patients, the current study allowed the evaluation of these individuals consistently. Of the thirty patients studied, only seven have secondary causes of hypertension, showing a prevalence of 23.3%.

 The score for renovascular disease was 8.6, which corresponds to an estimated frequency of approximately 9% according to the diagram of Drastic study [4]. This estimated prevalence is similar to the frequency of renovascular disease observed in this study (6.6%). 

 In the current study, of the thirty patients evaluated, 13 showed high risk for obstructive sleep apnea, which represents prevalence of 43.3%. In a recent study, Calhoun and cols [10] suggested that there is a close relationship between the serum level of aldosterone and obstructive sleep apnea. According to these authors, elevated aldosterone levels correlate with greater severity of apnea. In the present study, both patients diagnosed with primary hyperaldosteronism presented a high risk for obstructive sleep apnea according to the Berlin questionnaire.

 Prospective studies have shown that the prevalence of primary hyperaldosteronism in patients with resistant hypertension reaches about 14%–21%, which is too high in relation to the general hypertensive population. In our study, two patients had this diagnosis representing 6.6% of the studied sample.

 In the set of patients studied, 68% did not have adequate compliance to drug treatment, highlighting that this is one of the main factors limiting the reduction of blood pressure in these patients. The lifestyle, associated with high sodium intake, a sedentary lifestyle, and obesity, is a factor that complicates even more the appropriate treatment of these patients.

 There was also high frequency of the use of nonsteroidal anti-inflammatory drugs. It is known that these drugs attenuate the antihypertensive effect of diuretics and converting enzyme inhibitors which may contribute to the resistance to treatment.

 Surprisingly, the frequency of alcoholism was low, only one patient, which can be explained by a possible selection bias with a female predominance. It is due to the knowledge and the awareness of women in comparison with that of men to seek medical care for their problems, so we can speculate that alcoholics with resistant hypertension could be underdiagnosed.

## 6. Conclusion

 The data obtained suggest that among patients with resistant hypertension, there is a higher prevalence of secondary hypertension than that observed in general hypertensive ones and higher prevalence of sleep apnea. But there are a predominance of obesity, noncompliance either with diet or drugs and frequent use of hypertensive drugs. These latter factors are likely approachable at primary level health care, since that detailed anamnesis directed to the causes of resistant hypertension are applied.
